# Prenatal Acoustic Signals Influence Nestling Heat Shock Protein Response to Heat and Heterophil-to-Lymphocyte Ratio in a Desert Bird

**DOI:** 10.3390/ijms252212194

**Published:** 2024-11-13

**Authors:** Eve Udino, Anaïs Pessato, BriAnne Addison, Ondi L. Crino, Katherine L. Buchanan, Mylene M. Mariette

**Affiliations:** 1 School of Life & Environmental Sciences, Deakin University, 75 Pigdons Road, Geelong, VIC 3216, Australia; 2 Max Planck Institute for Biological Intelligence, Eberhard-Gwinner-Straße, 82319 Seewiesen, Germany; 3 Centre d’Écologie et des Sciences de la Conservation (CESCO), Muséum National d’Histoire Naturelle, Centre National de la Recherche Scientifique, Sorbonne Université, CP 135, 57 rue Cuvier, 75005 Paris, France; 4 College of Science and Engineering, Flinders University, Adelaide, SA 5042, Australia; 5 Doñana Biological Station EBC-CSIC, Calle Américo-Vespucio 26, 41092 Sevilla, Spain

**Keywords:** acoustic developmental programming, glucocorticoid, leucocyte profile, phenotypic plasticity, prenatal sound, stress proteins, *Taeniopygia guttata*

## Abstract

Heat shock proteins (HSPs) are essential to cellular protection against heat stress. However, the causes of inter-individual variation in HSP regulation remain unclear. This study aimed to test the impact of early-life conditions on the HSP response to heat in zebra finches. In this arid-adapted bird, incubating parents emit “heat-calls” at high temperatures, which adaptively alter offspring’s phenotypes. Embryos were exposed to heat-calls or control-calls, and at 13 days post-hatch nestlings were separated into two different experiments to test responses to either chronic nest temperature (“in-nest” experiment) or an acute “heat-challenge”. Blood samples were collected to measure levels of heat shock cognate 70, heat shock protein 90α, corticosterone and the heterophil-to-lymphocyte (H/L) ratio. In the in-nest experiment, both HSPs were upregulated in response to increasing nest temperatures only in control-calls nestlings (HSC70: *p* = 0.010, HSP90α: *p* = 0.050), which also had a marginally higher H/L ratio overall than heat-call birds (*p* = 0.066). These results point to a higher heat sensitivity in control-call nestlings. Furthermore, comparing across experiments, only the H/L ratio differed, being higher in heat-challenged than in in-nest nestlings (*p* = 0.009). Overall, this study shows for the first time that a prenatal acoustic signal of heat affects the nestling HSP response to postnatal temperature.

## 1. Introduction

Animals respond to environmental fluctuations by adjusting their physiology to current conditions. In particular, environmental challenges such as changes in weather, predator presence or food scarcity trigger a complex and multi-level “stress response”, which allows animals to maintain homeostasis in the face of such stressors [1,2]. Understanding organism responses to stressors is essential, because these responses define the fitness consequences of environmental disturbances, and thus ultimately the impact of disturbances on population persistence [3,4]. However, it remains unclear how individuals vary in their stress responses, from the cellular to organismal levels, and what factors may contribute to such inter-individual variation [2,5].

Within cells, heat shock proteins (HSPs) are essential molecular chaperones that play important functions to maintain proteostasis (i.e., protein homeostasis) and, therefore, cell survival under stress [6,7]. These highly conserved molecules respond to multiple stressors, including heat stress [8,9,10]. In particular, HSP70 and HSP90 families are well studied for their role in thermotolerance [11,12,13]. The stress-inducible HSPs (e.g., HSP70, HSP90α) are upregulated under acute stress [12,13], while constitutive HSPs (e.g., heat shock cognate 70, HSC70) are always expressed but modulated by long-term environmental conditions, such as thermal acclimatization (reviews: [4,10]; studies: [14,15]). Overall, however, compared to the wealth of studies on the molecular mechanisms of HSP regulation in vitro or under controlled laboratory conditions, much less is known about HSP regulation in an ecological context and its role in adaptation to environmental stressors [2,16].

At the whole-organism level, acute environmental stressors trigger the so-called “stress response”, which is mediated by the activation of the hypothalamic–pituitary–adrenal (HPA) axis and the subsequent release of glucocorticoid (GC) hormones into the peripheral blood within minutes [17,18]. These hormones bind primarily to glucocorticoid receptors (GR) within cells, whose maturation and functioning involves the participation of HSP70 and HSP90, respectively [19,20]. This suggests HSPs may also modulate the impact of GC release on the organism, at least in mammals [19,20]. Furthermore, across taxa, when exposure to a stressor or elevated GC levels becomes chronic, the relative proportion of circulating white blood cells in peripheral blood is altered [21,22]. Namely, heterophil numbers (in birds, or neutrophil in mammals) increase while lymphocyte numbers decrease, resulting in an elevation of the heterophil-to-lymphocyte (H/L) ratio [23,24]. The H/L ratio is a robust biomarker of medium-to-long-term stress because its time course is slower, steadier and longer lasting than the acute GC response [25,26]. Considering these organismal responses may help with interpreting inter-individual differences in HSP regulation.

Variability in HSP responses is observed between species [4,13], and between populations that differ in environmental harshness or predictability (e.g., [14,27]). However, less is known about how much and why HSP responses vary between individuals. Heritability of the HSP response is moderate (e.g., turtle: [28]; drosophila: [29]; human: [30]) and therefore only explains part of inter-individual variation. Early-life conditions, based on their well-known impact (especially for maternal GCs) on the organismal stress response and HPA axis regulation across taxa (including humans) [31,32], may also contribute to HSP variation. Indeed, at least in ectotherms, maternal HSP levels have been shown to increase offspring HSP levels and/or heat tolerance, (e.g., drosophila: [33]; zebrafish: [34]). In addition, incubation temperature in oviparous species may shape the HSP response (already functional during embryogenesis [28,35]). Specifically in chicken, controlled heat exposure during incubation can increase individual HSP levels during embryogenesis, but later decrease HSP levels and oxidative stress post-hatching during a heat-challenge [36]. However, exposure to high temperatures at the juvenile stage in zebra finches (*Taeniopygia guttata castanotis*) had a limited effect on HSP responses in adulthood [37]. Overall, therefore, while there is evidence that it can happen, the impact of early life experience on HSP responses remains little understood.

Among all organisms, birds are particularly exposed to high thermal extremes from being diurnal and not using underground thermal refuges [38,39]. Although the great majority of avian research focuses on poultry, there is evidence for the impact of high temperatures on HSP regulation (poultry: [11,12]; other birds: [13,40]), CORT levels [24,41] and the H/L ratio [42,43]. Nonetheless, high heat exposure in birds may also have favored specific adaptations to withstand heat, including at the cellular level [2]. This is the case in the zebra finch, a desert-adapted species, in which a prenatal acoustic signal of heat programs offspring development for high temperatures [44]. Zebra finch parents produce specific “heat-calls” during incubation at high temperatures, which adaptively shapes offspring growth to hot conditions [44], as well as nestling cellular metabolism and its response to temperature [45]. These changes led in adulthood to higher reproductive success [44] and generally lower chronic stress levels (measured as the H/L ratio) [46]. Prenatal heat-call exposure also led to higher heat tolerance in adulthood, although not through improved evaporative cooling efficiency [47]. Whether changes in HSP regulation could underlie some of these effects remains to be determined.

**Figure 1:**
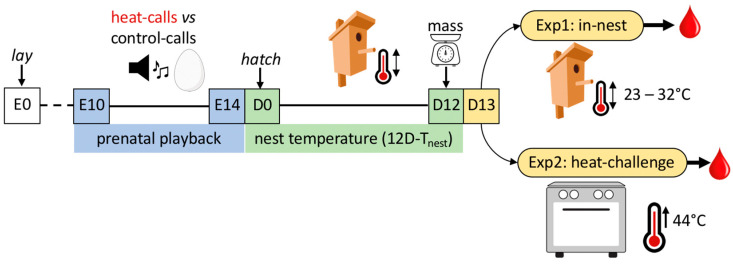
Experimental design: eggs were collected on the day of lay (embryonic day 0, E0) and artificially incubated until hatching (day 0, D0). Prenatal playback exposure (in blue) to either heat-calls or control-calls occurred in the last 5 days before hatching (E10 to E14), and the postnatal nest temperature (in green) was manipulated daily from D0 to D12 post-hatch. Nestlings’ body masses were measured on D12, and on D13 nestlings were separated into two experiments (in yellow), and a blood sample collected, to test the impact of prenatal acoustic experience on either (i) Experiment 1 (Exp1) in-nest: chronic response to nest temperature, or (ii) Experiment 2 (Exp2) heat-challenge: acute response to a heat-challenge in a temperature-controlled chamber.

Therefore, this study tested the hypothesis that early life conditions in a wild bird species influence HSP regulation later in life, in a way that may reflect changes towards heat adaption. Specifically, it tested whether prenatal exposure to heat-calls in the zebra finch affects individual HSP responses to chronic and acute thermal conditions, in parallel to other components of nestling stress physiology (CORT and the H/L ratio). To this aim, embryos were exposed to a prenatal playback of either heat-calls (treatment) or control-calls (control), and after hatching postnatal nest temperatures were manipulated. At 13 days post-hatch, the impact of early acoustic experience on responses to either chronic or acute thermal conditions were investigated in two separate experiments. Specifically, (i) in the “in-nest” experiment, nestlings were subjected to manipulated chronic nest temperatures (average daytime temperature from hatching to day 12, 12D-T_nest_, ranging from 23 to 32 °C), or, (ii) in the acute heat-challenge experiment, to a 2.5 h gradual temperature increase up to 44 °C for 20 min in a temperature-controlled chamber (Figure 1). Blood samples were then collected within three minutes of nest disturbance or at the end of the heat-challenge, to measure protein levels of the constitutive heat shock cognate 70 (HSC70) and stress-inducible HSP90α (in red blood cells), baseline CORT (in plasma) and the H/L ratio (from blood smears). We predicted that if prenatal heat-calls affect subsequent HSP regulation and prepare individuals for high temperatures in postnatal life, then in the in-nest experiment (i), a higher 12D-T_nest_ will be chronically challenging for control nestlings, but not for heat-call nestlings. Thus, an increase in both HSPs and both stress biomarkers with 12D-T_nest_ is expected in control-call birds only. In addition, in the heat-challenge experiment (ii), we predicted that control-call nestlings will be more affected by the heat-challenge than heat-call nestlings, resulting in higher stress-inducible HSP90α levels and H/L ratios (but not higher HSC70 [constitutively expressed] nor CORT [back to baseline levels after acute response: [25]]). Then, (iii) comparing across experiments, we expected that heat-challenged nestlings may have higher HSP90α levels and H/L ratios than in-nest nestlings, but similar levels of HSC70 and CORT. Lastly, (iv) given that HSP regulation may affect growth [9], we assessed how nestling mass relates to the stress biomarkers.

## 2. Results

### 2.1. Nestling Response to Nest Temperature: In-Nest Experiment

**Figure 2:**
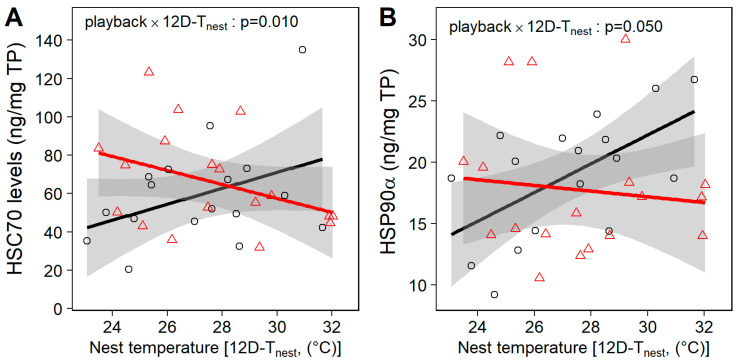
Levels of heat shock proteins across nest temperature in in-nest nestlings. Individuals were exposed to prenatal control-calls (black circles) or heat-calls (red triangles) and, upon hatching until day 12, experienced a mean daytime nest temperature (12D-T_nest_). At day 13, the nestlings were taken from their nest to collect blood samples. (**A**) Levels of constitutive heat shock cognate 70 (HSC70; N = 35) and (**B**) stress-inducible heat shock protein 90α (HSP90α; N = 35) were measured in the red blood cells. Open circles show individual data and regression lines are represented with 95% CIs.

Under “in-nest” conditions, there was a significant interaction between the prenatal playback and the acclimatization or chronic temperature experienced by nestlings in the nest (i.e., 12D-T_nest_, averaged from hatching to day 12) on heat shock protein levels (HSC70: *p* = 0.010, HSP90α: *p* = 0.050; Table 1, Figure 2). Specifically, levels of both the constitutive HSC70 and stress-inducible HSP90α increased with 12D-T_nest_ in control-call nestlings, whereas in heat-call nestlings HSC70 levels decreased slightly with 12D-T_nest_, and HSP90α levels did not vary (Figure 2). This conforms to our prediction that hotter nests are more challenging for control-call birds and trigger an HSP upregulation.

**Figure 3:**
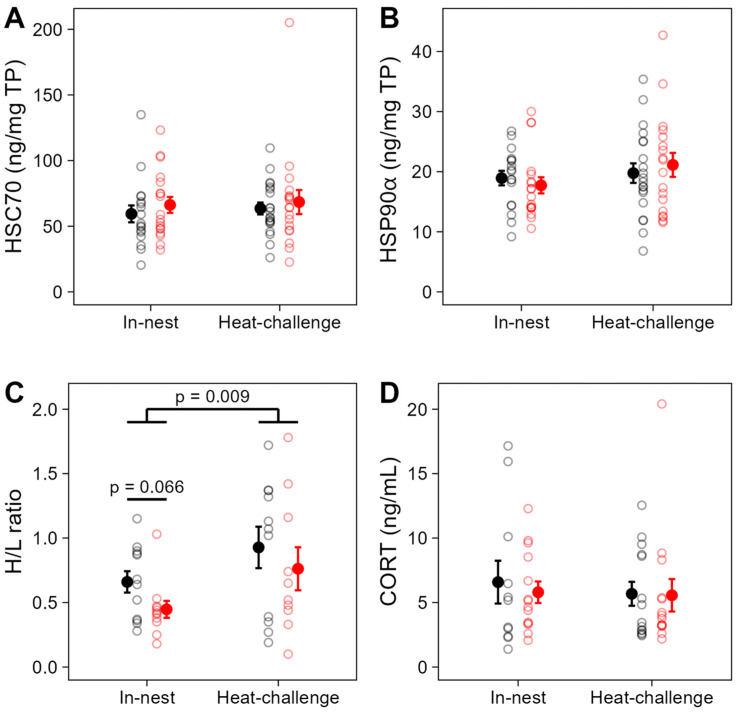
Means (±SE) of the stress biomarkers in in-nest or heat-challenged nestlings. Individuals were exposed to prenatal control-calls (black circles) or heat-calls (red circles) and, at day 13 post-hatch, blood samples were collected from individuals in their nest (in-nest) or following a heat-challenge. The panels show levels of (**A**) heat shock cognate 70 (HSC70; N = 73), (**B**) heat shock protein 90α (HSP90α; N = 73), (**C**) the heterophil-to-lymphocyte (H/L) ratio (N = 44) and (**D**) corticosterone (CORT; N = 53). Removing the high HSC70 value (**A**) or the high CORT value (**D**) did not change the results. Open circles show individual data and solid circles show group means ± SE.

The H/L ratio in in-nest nestlings was also affected by the prenatal playback, but not by nest temperature (Table 1). Indeed, the H/L ratio was marginally higher in control-call than heat-call nestlings (*p* = 0.066, Table 1, Figure 3C) across nest temperatures (no interaction playback by 12D-T_nest_; *p* = 0.283, Table S1). As for HSPs, this may indicate that the range of summer nest temperatures was overall more challenging for control nestlings.

By contrast, baseline CORT levels (taken within 3 min of disturbance) did not vary with prenatal acoustic experience (*p* = 0.860, Table 1, Figure 3D), nor nest temperature (i.e., no effect of 12D-T_nest_, *p* = 0.860, Table 1), including for control-call birds (no significant interaction playback by 12D-T_nest_; *p* = 0.997, Table S1). This may be in part because thermal conditions in mid-morning at the time of sampling were never extreme.

Lastly, nestling mass was not explained by any of the four biomarkers under in-nest conditions (all *p* > 0.151, Table 2).

### 2.2. Nestling Stress Response to a Controlled Acute Heat-Challenge

After a 2.5 h heat-challenge in a temperature-controlled chamber, contrary to our prediction, HSP90α levels were overall not higher than under in-nest conditions (*p* = 0.344, Table 3, Figure 3B), possibly due to the relatively mild, but ecologically realistic, heating procedure we used (progressive temperature increase to a maximum of 44 °C for 20 min). HSC70 and CORT levels were also similar to those in in-nest nestlings, as predicted (*p* > 0.596, Table 3, Figure 3A,D). Nonetheless, the H/L ratio was significantly higher after the heat-challenge, as expected (*p* = 0.009, Table 3, Figure 3C).

Within the heat-challenge experiment, the prenatal playback and nest temperature that nestlings had previously experienced (12D-T_nest_) did not affect any of the stress biomarkers at the end of the heat-challenge (for all, *p* > 0.557, Table 1, Figure 3). This suggests that developmental conditions did not explain inter-individual variations in response to an acute heat-challenge.

**Figure 4:**
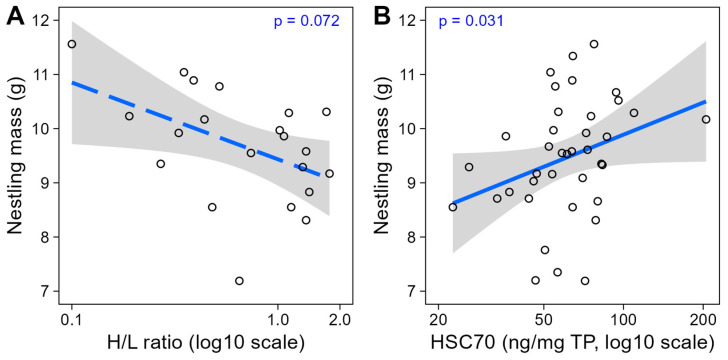
Body mass of heat-challenged nestlings in relation to their (**A**) heterophil-to-lymphocyte (H/L) ratio (N = 21) and (**B**) heat shock cognate 70 (HSC70) levels (N = 38). In (**B**), the *p*-value is marginal (*p* = 0.051) when the high HSC70 value is excluded. Regression lines are represented with 95% CIs.

Instead, biomarker levels under acute heat exposure were weakly related to nestling mass at day 12 (a proxy for individual “quality” or condition). Namely, a lower H/L ratio after a heat-challenge tended to occur in heavier nestlings (*p* = 0.072, Table 2, Figure 4), which points to a possibly lower sensitivity to acute heat in individuals in better body condition. Nonetheless, after the heat-challenge, HSC70 levels tended to be higher in heavier nestlings (*p* = 0.031, Table 2, Figure 4), although this effect was potentially only driven by one individual with high HSC70 levels (linear model result without the outlier: t = 2.03, *p* = 0.051; Figure 4B).

Lastly, as expected based on the different time courses of the various responses [25,48], we found that the stress biomarkers were not correlated to each other, considering each experiment separately or together (Table S2).

## 3. Discussion

This study is the first to demonstrate that prenatal acoustic experience affects the heat shock protein (HSP) response to thermal environment. In addition to maternal ovarian HSPs or incubation temperature [34,36], this study therefore reveals a novel parental mechanism that leads to alterations in HSP levels in their offspring. It shows that a prenatal acoustic signal of heat affects physiological responses to postnatal chronic temperatures in zebra finch nestlings. While hot nest conditions were usually not extreme for this species (mean daytime temperature < 32 °C; maximum temperature reached daily ranging from 18.0 to 43.4 °C), they were more challenging for nestlings (i.e., the control-call group), which had not received prenatal anticipatory cues about upcoming hot conditions. Specifically, control-call individuals had higher levels than heat-call individuals of both the constitutive heat shock cognate 70 (HSC70) and stress-inducible HSP90α in hotter nests, and a marginally higher heterophil-to-lymphocyte (H/L) ratio overall. This study thereby demonstrates that prenatal acoustic experience contributes to biologically relevant inter-individual variation in HSP regulation. By contrast, corticosterone (CORT) levels, even though they varied between individuals, were not related to prenatal acoustic experience or current thermal conditions in either experiment. This highlights the value of considering multiple stress biomarkers in parallel, to gain better insights into the physiological impact of environmental fluctuations. Overall, our study suggests that a prenatal acoustic signal of heat may improve nestling resilience to chronically high postnatal temperatures.

### 3.1. Acoustic Programming of HSPs and Stress Physiology

This study found that prenatal sound was capable of altering nestling stress physiology two weeks later, in response to chronic nest temperature. This aligns with previous research on the zebra finch showing that prenatal heat-call exposure leads to inter-individual variation later in life, specifically in traits related to thermoregulation and heat responses (e.g., [44,45,47,49]), but not in other traits (e.g., foraging skills [50]). This study therefore adds to the emerging evidence for the role of prenatal sound in developmental programming, with both positive and negative fitness consequences [44,51,52]. Even though the fitness impact was not measured here, differences in HSP levels are likely to reflect an advantage for heat-call birds under chronic heat. Indeed, in hot nests protein levels for both HSPs were higher in control-call birds than in heat-call birds, which is potentially indicative of higher protein unfolding triggering the HSP upregulation. By contrast, heat-call individuals in hot nests presumably invested less energy into HSP synthesis, leaving more energy for other functions (e.g., the immune system [53]), or lowering overall energy requirements in the heat when the parental provisioning rate is reduced [54,55]. While the molecular mechanisms leading from prenatal sound exposure to differential HSP responsiveness in nestlings are unknown, it is likely that this effect occurred through an indirect pathway, via changes in individual thermal physiology. One previous study nonetheless reported that loud prenatal music increased HSP levels in chicken embryos and hatchlings [56], which also points to an acoustic sensitivity of HSP regulation and possibly other cellular processes [57].

In agreement with HSP level differences reflecting different sensitivities to heat, we found that heat-calls birds had a marginally lower H/L ratio than control-call nestlings in summer. This is consistent with results in adult zebra finches (from a different cohort), where H/L ratios were also lower in heat-call birds in summer, but also, surprisingly, in winter [46]. Both of these results, in nestlings and adults, therefore may indicate a higher resilience of heat-call individuals to thermal fluctuations, rather than solely to hot conditions. This may be related to the ecological conditions in the Australian desert, where heatwaves can trigger storms associated with a drop in temperature. Lastly, unlike in yellow-legged gulls where alarm calls signaling predator presence tends to lead to higher baseline CORT levels at hatching [58,59], we found no effect of prenatal acoustic experience on baseline CORT levels, possibly because nest temperatures were always mild at the time of sampling (in the late morning).

### 3.2. HSP Response to Acute and Chronic Heat

In individuals that had not received the prenatal acoustic signal of heat (i.e., control-call birds), a high chronic temperature in the nest led to increased levels of both HSP90α and HSC70. Even though HSC70 is generally classified as a constitutive HSP, there is evidence that gene expression for HSC70 (*HSPA8*) can be upregulated in the heat, although less readily so than *HSP90-B1* [13,35,60]. Against our expectations, however, the heat-challenge in our second experiment did not trigger an increase in either HSP compared to nestlings in the first experiment. This may be because, rather than a sudden increase to an extreme temperature lasting several hours, we used a staggered temperature increase (starting at 27 °C), with the most extreme temperature stage (at 44 °C) only lasting 20 min and ending 18 min before blood sampling. This protocol, which represents a more natural heat exposure, may not have triggered significant protein unfolding (which would trigger the HSP upregulation [11]) or it may not have allowed sufficient time to detect HSP upregulation at the protein levels (which takes longer than HSP gene upregulation). The time course of the HSP response, especially for protein translation, is still unclear in birds [11,40], partly because results on cell cultures [61,62], which lack organismal regulatory pathways, may not directly extrapolate to the whole-organism kinetic. Nonetheless, our results may also be indicative of a lower sensitivity of zebra finches to acute heat than other species. For example, in three Australian desert birds, HSP gene expression was upregulated in all species, but the extent of the response across HSP genes and organs was much lower in the zebra finch than in the diamond dove (*Geopelia cuneate*) or budgerigar (*Melopsittacus undulates*) [13].

### 3.3. Stress Response to Acute Heat

Exposure to the acute heat-challenge nonetheless increased the H/L ratio, as also found in adults in two other desert species [24] and in poultry [42,43,63]. In addition, nestlings with a lower body mass tended to have a higher H/L ratio, as also reported in adults, in the zebra finch [46] and other avian species (e.g., [64]), although this was only seen in the heat-challenge, and not in in-nest conditions. This may indicate a higher sensitivity of smaller nestlings to acute heat but not to long-term high temperatures. Furthermore, CORT levels from heat-challenged nestlings did not differ from those sampled in in-nest conditions. Even if heat itself did not increase CORT levels, handling just before the heat-challenge would have triggered a CORT response, as observed in other studies on zebra finch nestlings exposed to a standard restraint protocol [65,66]. The similar CORT levels across the two experiments therefore do not indicate that nestlings in the heat treatment did not experience stress, but rather that their CORT levels had returned to baseline by the end of the 2.5 h heat-challenge, which is consistent with the expected time course of CORT in response to an acute stressor [48]. This short response may explain why previous studies also failed to find a CORT increase after an acute heat-challenge [24], or during heatwaves in the wild [67].

### 3.4. Stress Biomarker Covariation

Overall, this study suggests that the response profiles of the organismal and cellular stress biomarkers provide complementary information on an individual’s physiological state. This is also indicated by the absence of correlation between the stress biomarkers. The lack of correlation between plasma CORT levels and the H/L ratio is common [24,25,68]. By contrast, to our knowledge, relationships between HSPs and CORT or the H/L ratio have been tested only in one previous study, which also reported an absence of correlation in blackbirds (*Turdus merula*) [69]. That, overall, we found differences in the H/L ratio and HSPs but not in CORT, supports the usefulness of measuring other stress biomarkers in addition to CORT, in order to better interpret the effect of a stressor or chronic conditions on individuals [70,71].

### 3.5. Conclusions

In conclusion, this study shows that prenatal exposure to “heat-calls”, an acoustic signal of heat, affects the stress physiology response of zebra finch nestlings to postnatal chronic temperatures. It showed that heat-call-exposed individuals did not upregulate heat shock proteins in hot nests, which, combined with lower H/L ratios, is indicative of a lesser sensitivity to high temperatures than control birds. This study therefore adds to the increasing evidence in this system showing that heat-call exposure shifts offspring traits towards a heat-adapted phenotype. It also adds to the small number of studies to date on the sources of inter-individual variation in HSP regulation in wildlife, and the role of developmental programming in generating such variation.

## 4. Materials and Methods

### 4.1. Prenatal Acoustic Playback

In this study, nestlings were obtained from the same breeding experiment described in [45]. The breeding experiment (see Figure 1 for a summary diagram), was carried out at Deakin University, Geelong, Australia, from January to April 2019. In total, 59 male and 52 female wild-derived zebra finches were allowed to pair and breed freely in an outdoor aviary (12 × 6 × 3 m). Eggs were collected on laying day and replaced with dummy eggs. Freshly collected eggs were incubated in a fan-less artificial incubator (Bellsouth 100 electronic incubator) at 37.5 °C and 60% humidity. On the 10th incubation day, eggs were randomly transferred into one of two experimental incubators until hatching, 4–5 days later, and were exposed daily to either heat-calls (treatment) or control-calls (control). Heat-calls are fast, high-pitch calls given by adult zebra finches under heat stress, whereas as control-calls, “tet-calls” were used, which are contact calls commonly uttered by parents at the nest, including during incubation, but which are not related to temperature [44,72]. Both groups were also exposed to whine calls, characterized by a complex acoustic structure, to ensure normal stimulation of the auditory system. Prenatal playbacks were broadcast from 10:00 to 18:00 at 65 dB from two speakers (Sennheiser HD439, Video Guys, Melbourne, Australia) inside the incubators and externally connected to an amplifier (Digitech 18 W, Jaycar, Geelong, Australia) and an audio player (Zoom H4nSP and Marantz PMD670, Video Guys, Melbourne, Australia). Egg trays and audio players were swapped daily between the two playback incubators to prevent incubator-specific effects. Upon hatching, nestlings were returned to foster parents and broods of three-to-four nestlings were composed from both prenatal playback groups.

### 4.2. Postnatal Nest Temperature

The nest temperature varied naturally with air temperature, and was, in addition, experimentally manipulated during daytime (10:00–18:00) throughout nestling development (from hatching to 13 days post-hatch) to obtain a gradient of cool-to-hot nests. Cool nests were provided shade using a shading cloth on the roof of the aviary and cool pads (for air temperature (T_a_) between 25 °C and 30 °C) or frozen pads (for T_a_ > 30 °C) placed under the nest-box roof. Hotter nests were provided heat pads: one placed under the nest-box roof (for T_a_ < 30 °C, Medi Heat Pack^®^, Melbourne, Australia), and a second one at the back of the nest, inside the nest-box (for T_a_ < 25 °C, Hotteeze^®^, Melbourne, Australia). All pads were placed at 10:00, replaced at 13:30 and 15:30, and removed at 18:00. The nest temperatures were continuously recorded with thermometers (Minnow-1.0TH, Senonics, Instrument Choice, Adelaide, Australia) placed in the nest boxes. The average nest temperature experienced by each nestling from hatching to 12 days post-hatch during daytime (11:00–18:00) was calculated, referred to as “12D-T_nest_”. 12D-T_nest_ per nestling varied from 23.1 to 33.8 °C. Within the daytime temperature variation, the highest nest temperature reached per day varied from 18.0 to 43.4 °C (mean = 30.0 °C).

### 4.3. In-Nest and Heat-Challenge Experiments

All individuals were weighed at 12 days post-hatch between 15:00 and 15:30 to determine their body mass. At day 13, nestlings were allocated to either the “in-nest” or “heat-challenge” experiment. In the in-nest experiment, nestlings were picked up from their nests, between 11.30 and 12.45, immediately prior blood sampling. In the heat-challenge experiment, nestlings were exposed to an acute heat-challenge. Specifically, they were picked up from their nests at 09:30 and food-deprived (for metabolic measurement) until blood sampling. They were acclimated at room temperature (25 °C) for 30 min, then at 29 °C in a temperature-controlled chamber. Then, they were exposed to 20–30 min stages at 35, 40, 42 and 44 °C in the chamber, before the temperature was dropped to 35 °C for 15 min. The heat-challenge lasted 2.5 h in total, except for two individuals showing distress signs that required the experiment to stop at 42 °C.

Within 3 min of collection from the nest or the end of the heat-challenge, nestlings were euthanized using isoflurane and decapitation to collect tissues as part of a separate study. Up to 250 µL of blood from the jugular vein was collected with heparinized capillary tubes, a blood smear was prepared (see below) and the remaining blood was expelled in microcentrifuge tubes. Blood samples were kept on ice for up to 30 min until centrifugation, at 2000× *g* for 10 min at 4 °C. Plasma and red blood cell (RBC) samples were stored at −80 °C until assayed for corticosterone (CORT) and heat shock proteins (HSPs). Up to two samples were collected per day, and, in total, 75 blood samples and smears were collected.

All procedures described in this study were approved by the Animal Ethics Committee of Deakin University (G23-2018) and were carried out in accordance with the Australian guidelines and regulations for the use of animals in research.

### 4.4. Corticosterone Assays

Corticosterone levels were quantified in 53 plasma samples using Enzyme Immunoassay (EIA) kits (ADI-901-097, Enzo Life Sciences, Farmingdale NY, USA). All samples were spiked with tritiated CORT (NET399250UC; Perkin Elmer, Sydney, Australia) to determine the recovery percentage. CORT was extracted twice from plasma using dichloromethane. After evaporation under nitrogen gas, samples were reconstituted in buffer solution (1:25 dilution). We ran the samples following an adjusted protocol using half the volume of the reagents provided in the EIA kit [72]. Every plate had a six-point standard curve ranging from 20,000 pg/mL to 6.4 pg/mL and an external CORT standard of 500 pg/mL to calculate the inter-plate variation. All samples and standards were run in triplicates. The plates were read using a Varioskan Lux microplate reader (Thermo Fisher Scientific, Melbourne, Australia) at 405 nm corrected at 580 nm. The CORT concentrations were corrected by the sample recoveries (the mean recovery was 69%). The intra- and inter-plate coefficients of variation were 4.15% and 9%, respectively.

### 4.5. HSPs Assays

Levels of HSC70 and HSP90α were quantified in 73 RBC samples using EIA kits (SKT-106, SKT-107, StressMarq Biosciences Inc., Victoria, BC, Canada) following the manufacturer’s instructions and a published protocol [40] that was optimized in the laboratory to our samples. For protein extraction, 2 µL of RBCs were diluted with 1× extraction reagent solution (1:500 dilution) containing protease inhibitors (Cat. No. 04693159001, Sigma Aldrich, Melbourne, Australia). The samples were incubated on ice for 30 min and vortexed for 10 s at T_0_, T_10_, T_20_ and T_30_ min. Then, the samples were centrifuged at 4500 RPM (4520× *g*) for 10 min at 4 °C. The supernatant was collected and stored at −80 °C until further processing.

Bradford assays [73] were then performed to quantify the total protein (TP) content of the samples. Briefly, 200 µL of Bradford reagent (B6916, Sigma-Aldrich) was added to 5 µL of the sample into the wells of a plate (NUN475094, Thermo Fisher Scientific). The standard curves were generated using a known concentration of bovine serum albumin (P0834, Sigma-Aldrich). Plates were incubated for 5 min at room temperature and read at 595 nm with a Varioskan Lux microplate reader.

To assay HSC70 and HSP90α, samples were run undiluted and diluted (1:2) with the sample diluent supplied in the kit, respectively. Every plate had a seven-point standard curve ranging from 150 ng/mL to 2.34 ng/mL for HSC70, and 28 ng/mL to 0.44 ng/mL for HSP90α, as well as an inter-assay standard to calculate inter-plate variation. All samples and standards were run in duplicate. The plates were read at 450 nm using a Varioskan Lux microplate reader. The HSP concentrations were corrected by TP content. The intra- and inter-plates variations were 7.7% and 12.9% for HSC70, and 8.7% and 25% for HSP90α.

### 4.6. Heterophil-to-Lymphocyte Ratio

Blood smears were prepared by smearing one drop of fresh blood on a glass slide to obtain a thin blood film. All smears were immediately air-dried and stained using Quick Dip staining (Fronine, Sydney, Australia). Specifically, the smears were fixed with the fixative solution (methanol) and dipped seven times successively into the two stain solutions (eosin Y and methylene azure B). Slides were rinsed (water), air-dried and stored in the dark at room temperature until analysis by a single observer (EU).

Leucocyte counts were performed using a compound microscope (Olympus Australia, Melbourne, Australia) at 1000× magnification using oil immersion. The first 100 leucocytes were counted, differentiating heterophils, eosinophils, basophils, lymphocytes and monocytes according to standard avian guidelines [74]. Then, the H/L ratio was calculated by dividing the number of heterophils by the number of lymphocytes. In total, leucocyte counts from 56 individuals were obtained.

To assess the repeatability of the measurements, a subset of 18 slides was examined a second time. Eight additional slides that showed an elevated H/L ratio (>3) were also counted a second time to verify if the first count was correct.

### 4.7. Statistical Analyses

All statistical analyses were conducted using R version 4.4.1. Physiological traits were not all measured in all individuals, and 13 blood smears were too small and/or thin to count 100 leucocytes, resulting in varying sample sizes: in the in-nest experiment, N = 25, 35, 35 and 28 birds for CORT, HSC70, HSP90α and the H/L ratio, respectively; in the heat-challenge experiment, sample sizes were 28, 38, 38 and 28 birds, respectively.

All models were fitted using the packages *lme4* (v 1.1-35-5) and *lmerTest* (v 3.1-3). Continuous predictors were scaled (i.e., mean = 0, std = 1), and the normality and homoscedasticity of residuals were visually inspected. Furthermore, collinearity among predictors was tested in the full models by calculating the variance inflation factors (VIFs), which were always lower than three, indicating no significant collinearity [75].

#### 4.7.1. H/L Ratio Distribution

While CORT and HSP levels were centered around the mean, the H/L ratio distribution was bimodal, with a great majority of nestlings with a ratio < 2 (i.e., 82% in the in-nest experiment [range: 0.18–1.15] and 75% in the heat-challenge experiment [0.10–1.78]) and a few individuals with high values > 2 (in-nest: 5 nestlings [2.22–6.91], heat-challenge: 7 nestlings [2.17–13.17]; Figure S1). These high H/L ratios were not the result of miscounts, as the H/L ratio, including for nestlings with high H/L ratios, was repeatable among the blood smears counted twice [intra-class correlations ICCs: N = 26, R = 0.73, CI = (0.49, 0.87), *p* = 0.001]. It is not known whether methodological issues other than cell identification and counting could lead to extreme H/L ratios. Although similarly extreme values in a few individuals have been reported in nestlings of another avian species [76], values > 2 were higher than any observed in zebra finch adults [46]. Given that the number of circulating heterophils and lymphocytes vary with other factors than stress [23,77,78], this suggests that nestlings with H/L ratios > 2 were experiencing an unknown challenge or condition (such as, for example, an ongoing bacterial infection), unrelated to the experimental manipulations. Accordingly, even after the heat-challenge, the H/L ratio distribution was still bimodal, which confirms that extreme H/L ratio values were unrelated to the experiment, and to the heat stress response. Given the uncertainty around the interpretation of these values, and since extreme values may mask or bias experimental effects and make data distribution less suitable for mixed models, H/L ratios > 2 were excluded from the analyses using the H/L ratio as a response variable. These individuals had, however, average levels of CORT and of both HSPs (Figure S2), which again points to a different driver than stress leading to extreme H/L ratios.

#### 4.7.2. Developmental Effects Within Each Experiment

First, for the experiment under in-nest conditions (chronic exposure to manipulated nest temperature), the effects of the developmental conditions (i.e., prenatal playback and postnatal nest temperature) were assessed on each stress biomarker after log-transformation (CORT, HSC70, HSP90α and H/L ratio). Linear mixed models (LMMs) were used, including the prenatal playback and nest temperature (12D-T_nest_) as predictors, and their interaction. Brood identity (rearing nest; “brood-ID”) was also included as a random factor, unless its variance was null (see Table S1). Brood size was not added as a covariate because brood size was standardized to four nestlings, and was consistently non-significant in preliminary analyses. Considering clutch identity (to account for genetic similarities) instead of brood identity as a random effect led to null or extremely low variance.

Second, for the acute heat-challenge experiment, developmental effects in heat-challenged individuals were tested, using the same models as for the in-nest experiment above. Expressing the nest temperature as the chamber temperature deviation from the nest temperature (i.e., chamber temperature above 12D-T_nest_: Δ12D-T_nest_, as in [45]) instead of the nest temperature did not change any of the results.

For all models, the interaction (playback × 12D-T_nest_) was removed when it was non-significant. Models with the interaction are reported in the Supplementary Materials (Table S1).

#### 4.7.3. Variation Across Experiments (In-Nest vs. Heat-Challenge)

To test whether stress biomarkers were affected by the heat-challenge, LMMs were run using each stress biomarker as the response variable, and the experiment (in-nest or heat-challenge) as a predictor, with brood identity (rearing nest; “brood-ID”) as a random factor, unless its variance was null (see Table 3).

#### 4.7.4. Link Between Nestling Mass and Stress Physiology

In each experiment, to test how the stress biomarkers may impact nestling mass, two separate LMMs were run, including mass as the response variable, brood-ID as a random factor and either CORT and the H/L ratio or both HSPs as predictors (log-transformed). Not all four stress biomarkers were included in a single analysis as this would considerably reduce the sample size because of missing values, although it led to the same results. As reported elsewhere [24,25,68,69], because of different response time courses, that the stress biomarkers were not correlated to each other was verified, considering each experiment separately or together (Table S2).

## Figures and Tables

**Table 1:** Linear mixed models ^1^ fitting levels of heat shock protein 90α (HSP90α), heat shock cognate 70 (HSC70), corticosterone (CORT) or the heterophil-to-lymphocyte (H/L) ratio as a function of the prenatal playback (heat-call or control-call), nest temperature (12D-T_nest_ ^2^) and their interaction in zebra finch nestlings.

Response Variable	Fixed Effect	Est. ^3^	SE ^4^	t Value	*p*-Value ^5^
In-nest condition					
HSP90α (N = 35) *	intercept	1.27	0.03	42.93	<0.001
	playback (heat-calls)	−0.04	0.04	−0.88	0.388
	**12D-T_nest_**	0.08	0.03	2.41	**0.022**
	**playback × 12D-T_nest_**	−0.09	0.04	−2.04	**0.050**
HSC70 (N = 35)	intercept	1.73	0.04	42.32	<0.001
	playback (heat-calls)	0.07	0.05	1.31	0.202
	12D-T_nest_	0.08	0.04	1.78	0.085
	**playback × 12D-T_nest_**	−0.15	0.06	−2.76	**0.010**
CORT (N = 25) *	intercept	0.68	0.09	7.58	<0.001
	playback (heat-calls)	0.02	0.12	0.18	0.860
	12D-T_nest_	0.01	0.06	0.18	0.860
H/L ratio (N = 23) *	intercept	−0.23	0.06	−3.93	0.001
	playback (heat-calls)	−0.16	0.08	−1.94	0.066
	12D-T_nest_	0.05	0.04	1.09	0.289
Heat-challenge condition					
HSP90α (N = 38)	intercept	1.26	0.04	33.62	<0.001
	playback (heat-calls)	0.02	0.05	25.14	0.656
	12D-T_nest_	0.01	0.03	20.75	0.799
HSC70 (N = 38) *	intercept	1.78	0.04	44.77	<0.001
	playback (heat-calls)	0.00	0.06	0.07	0.949
	12D-T_nest_	−0.02	0.03	−0.59	0.557
CORT (N = 28) *	intercept	0.68	0.07	9.72	<0.001
	playback (heat-calls)	−0.03	0.10	−0.27	0.789
	12D-T_nest_	−0.02	0.05	−0.49	0.632
H/L ratio (N = 21)	intercept	−0.10	0.10	−1.057	0.305
	playback (heat-calls)	−0.07	0.14	−0.512	0.615
	12D-T_nest_	0.04	0.08	0.485	0.634

* The random factor brood identity (brood-ID) was removed because its variance was null. ^1^ full model: response ~ prenatal playback + 12D-T_nest_ + playback × 12D-T_nest_ + (1|brood-ID); ^2^ 12D-T_nest_: mean daytime nest temperature experienced from hatching to day 12; ^3^ Est.: estimate; ^4^ SE: standard error; ^5^ *p*-values below 0.05 (alpha level threshold) are significant and indicated in bold.

**Table 2:** Full linear models of nestling mass as a function of heat shock cognate 70 (HSC70) and heat shock protein 90α (HSP90α), or corticosterone (CORT) and the heterophil-to-lymphocyte (H/L) ratio.

Physiological Predictor	Fixed Effect	Est. ^1^	SE ^2^	t Value	*p*-Value ^3^	Variance [brood-ID ^4^] (±SE)
In-nest condition						
HSC70 and HSP90α (N = 35)	Intercept	9.26	0.21	43.82	<0.001	0.722 (±0.850)
	HSC70	−0.27	0.18	−1.48	0.151	
	HSP90α	0.04	0.17	0.26	0.801	
CORT and H/L ratio (N =17)	Intercept	9.52	0.28	34.14	<0.001	0.674 (±0.822)
	CORT	0.20	0.26	0.75	0.464	
	H/L ratio	−0.29	0.23	−1.24	0.252	
Heat-challenge condition						
HSC70 and HSP90α (N = 35)	Intercept	9.45	0.17	55.55	<0.001	0.121 (±0.348)
	**HSC70**	0.37	0.17	2.25	**0.031**	
	HSP90α	−0.28	0.17	−1.64	0.109	
CORT and H/L ratio (N = 17)	Intercept	9.68	0.26	37.80	<0.001	null (removed)
	CORT	−0.02	0.28	−0.09	0.930	
	H/L ratio	−0.54	0.28	−1.95	0.072	

^1^ Est.: estimate; ^2^ SE: standard error; ^3^ *p*-values below 0.05 (alpha level threshold) are significant and indicated in bold; ^4^ brood-ID: brood identity.

**Table 3:** Linear models ^1^ fitting each stress biomarker of zebra finch nestlings as a function of the experiment (in-nest or heat-challenge) in a pooled dataset.

Response Variable	Fixed Effect	Est. ^2^	SE ^3^	t	*p*-Value ^4^	Variance [brood-ID ^5^] (±SE)
CORT ^6^ (N = 53)	intercept	0.70	0.05	12.92	<0.001	null (removed)
	experiment (heat-challenge)	−0.03	0.07	−0.37	0.712	
HSC70 ^7^ (N = 73)	intercept	1.76	0.03	58.26	<0.001	0.003 (±0.05)
	experiment (heat-challenge)	0.02	0.04	0.53	0.596	
HSP90α ^8^ (N = 73)	intercept	1.24	0.03	48.75	<0.001	null (removed)
	experiment (heat-challenge)	0.03	0.04	0.95	0.344	
H/L ratio ^9^ (N = 44)	intercept	−0.33	0.06	−5.70	<0.001	0.03 (±0.19)
	**experiment (heat-challenge)**	0.21	0.08	2.76	**0.009**	

^1^ model: response ~ experiment + (1|brood-ID); ^2^ Est.: estimate; ^3^ SE: standard error; ^4^ *p*-values below 0.05 (alpha level threshold) are significant and indicated in bold; ^5^ brood-ID: brood identity; ^6^ CORT: corticosterone; ^7^ HSC70: heat shock cognate 70; ^8^ HSP90α: heat shock protein 90α; ^9^ H/L ratio: heterophil-to-lymphocyte ratio.

## Data Availability

The data supporting reported results are publicly available on the Mendeley repository: https://doi.org/10.17632/mrc9yzngwm.1.

## Supplementary Materials

The following supporting information can be downloaded at https://www.mdpi.com/article/10.3390/ijms252212194/s1.
