# Supplementary material for: Prenatal Acoustic Signals Influence Nestling Heat Shock Protein Response to Heat and Heterophil-to-Lymphocyte Ratio in a Desert Bird
Source: Int J Mol Sci. 2024 Nov 13;25(22):12194. doi: 10.3390/ijms252212194 (PMC11595141; doi:10.3390/ijms252212194)
Supplement: Supplementary file 1 [file ijms-25-12194-s001.zip › ijms-3233916-supplementary.pdf]

Supplementary materials for

Prenatal acoustic signals influence nestling heat-shock protein response to heat and heterophil-to-lymphocyte ratio in a desert bird

Eve Udino*, Anaïs Pessato, BriAnne Addison, Ondi L. Crino, Katherine L. Buchanan, and Mylene M. Mariette*

*Correspondence: m.mariette@deakin.edu.au, eve.udino@bi.mpg.de

This file contains:

- Table S1 2
- Table S2 3
- Figure S1 4
- Figure S2 5

Table S1. Full models[1] (including interaction) of CORT, HSC70 and HSP90α levels, and H/L ratio, as function of the prenatal playback (heat-call or control-call), mean daytime nest temperature experienced from hatching to 12-day (12D-T_{nest}), and their interaction, in zebra finch nestlings. Bold indicates significant p-values <0.05.

Response variable	Fixed effect	Est.	SE	t	p-value	variance [brood id] (±SE)
In-nest conditions						
CORT (n = 25)	Intercept	0.68	0.09	7.39	<0.001	null (removed)
	playback (heat-call)	0.02	0.12	0.17	0.863	
	12D-T_{nest}	0.01	0.09	0.12	0.903	
	playback x 12D-T_{nest}	0.00	0.13	0.00	0.997	
HSC70 (n = 35)	Intercept	1.73	0.04	42.32	<0.001	0.01 (±0.12)
	playback (heat-call)	0.07	0.05	1.31	0.202	
	12D-T_{nest}	0.08	0.04	1.78	0.085	
	**playback x 12D-T_{nest}**	-0.15	0.06	-2.76	**0.010**	
HSP90α (n = 35)	Intercept	1.27	0.03	42.93	<0.001	null (removed)
	playback (heat-call)	-0.04	0.04	-0.88	0.388	
	**12D-T_{nest}**	0.08	0.03	2.41	**0.022**	
	**playback x 12D-T_{nest}**	-0.09	0.04	-2.04	**0.050**	
H/L ratio (n = 23)	Intercept	-0.22	0.06	-3.92	0.001	null (removed)
	playback (heat-call)	-0.16	0.08	-1.94	0.067	
	12D-T_{nest}	0.01	0.05	0.164	0.871	
	playback x 12D-T_{nest}	0.10	0.09	1.10	0.283	
Heat-challenge condition						
CORT (n = 28)	Intercept	0.68	0.07	9.53	<0.001	null (removed)
	playback (heat-call)	-0.03	0.10	-0.25	0.805	
	12D-T_{nest}	0.03	0.06	0.48	0.639	
	playback x 12D-T_{nest}	-0.01	0.11	-0.11	0.913	
HSC70 (n = 38)	Intercept	1.78	0.04	44.10	<0.001	null (removed)
	playback (heat-call)	0.00	0.06	0.08	0.939	
	12D-T_{nest}	0.02	0.04	0.46	0.646	
	playback x 12D-T_{nest}	0.00	0.06	-0.07	0.947	
HSP90α (n = 38)	Intercept	1.26	0.04	31.71	<0.001	0.01 (±0.12)
	playback (heat-call)	0.03	0.05	0.48	0.636	
	12D-T_{nest}	0.00	0.04	0.00	0.998	
	playback x 12D-T_{nest}	-0.03	0.05	-0.60	0.553	
H/L ratio (n = 21)	Intercept	-0.11	0.10	-1.032	0.317	0.06 (±0.25)
	playback (heat-call)	-0.07	0.14	-0.492	0.629	
	12D-T_{nest}	0.03	0.10	0.317	0.755	
	playback x 12D-T_{nest}	0.02	0.15	0.107	0.916	

[1]: full model: response ~ prenatal playback + 12D-T_{nest} + prenatal playback x 12D-T_{nest}

Table S2. Correlation matrix of CORT, HSC70 and HSP90α levels, and, H/L ratio in in-nest, heat-challenged zebra finch nestlings, and both experiments pooled. Values above diagonal indicate Pearson's correlation coefficients *r*, and values below diagonal sample sizes. All p-values range between 0.204 - 0.963.

**In-nest**	CORT	HSC70	HSP90α	H/L ratio
CORT		0.13	-0.14	0.10
HSC70	23		0.31	-0.01
HSP90α	23	27		-0.04
H/L ratio	20	27	27	
**Heat-challenge**				
CORT		0.04	0.10	0.08
HSC70	28		0.12	-0.15
HSP90α	28	28		-0.11
H/L ratio	22	28	28	
**Pooled datasets**				
CORT		0.01	-0.05	-0.12
HSC70	51		0.08	-0.02
HSP90α	51	55		-0.10
H/L ratio	42	55	55	

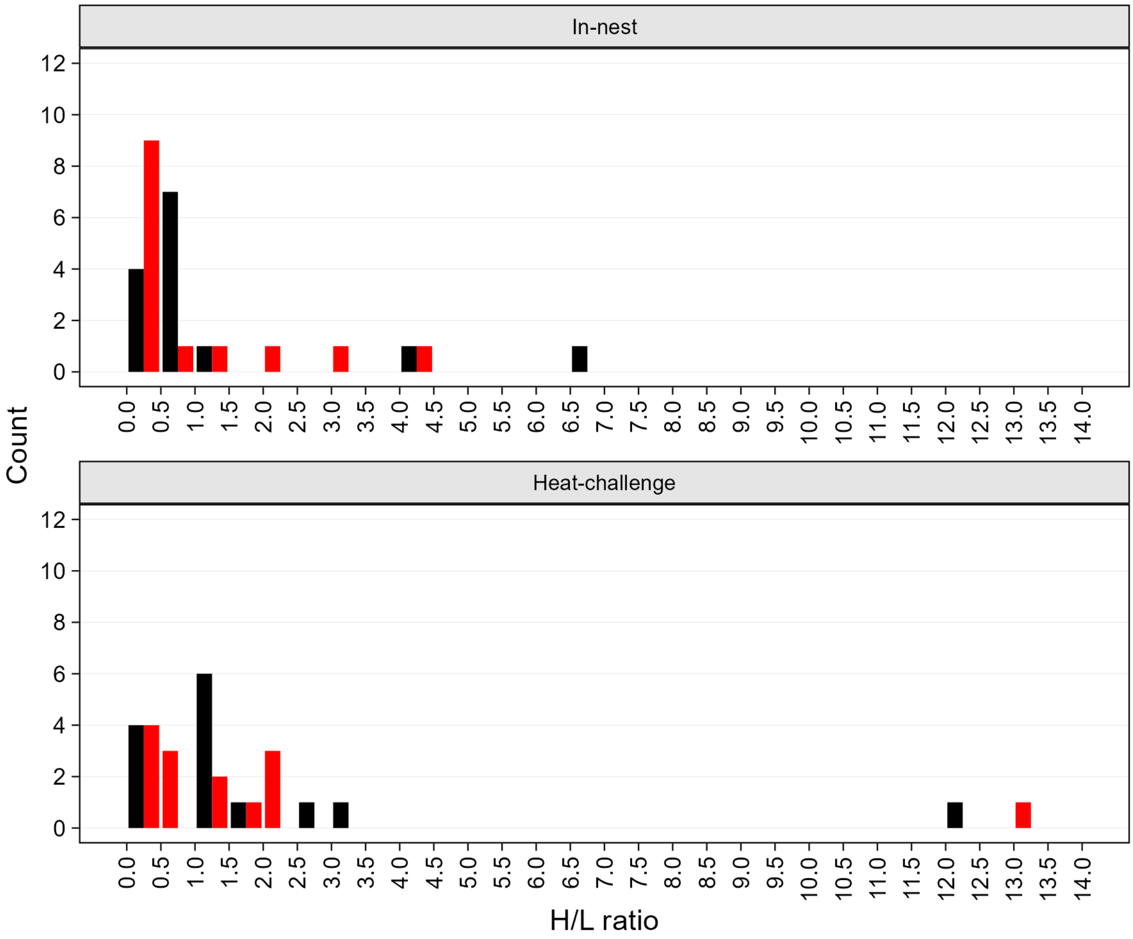

Figure S1. Histograms of the H/L ratio distribution in in-nest (N = 28) and heat-challenge (N = 28) individuals and across prenatal playback groups (black: control-calls, red: heat-calls). Histogram bars are dodged to prevent overlapping.

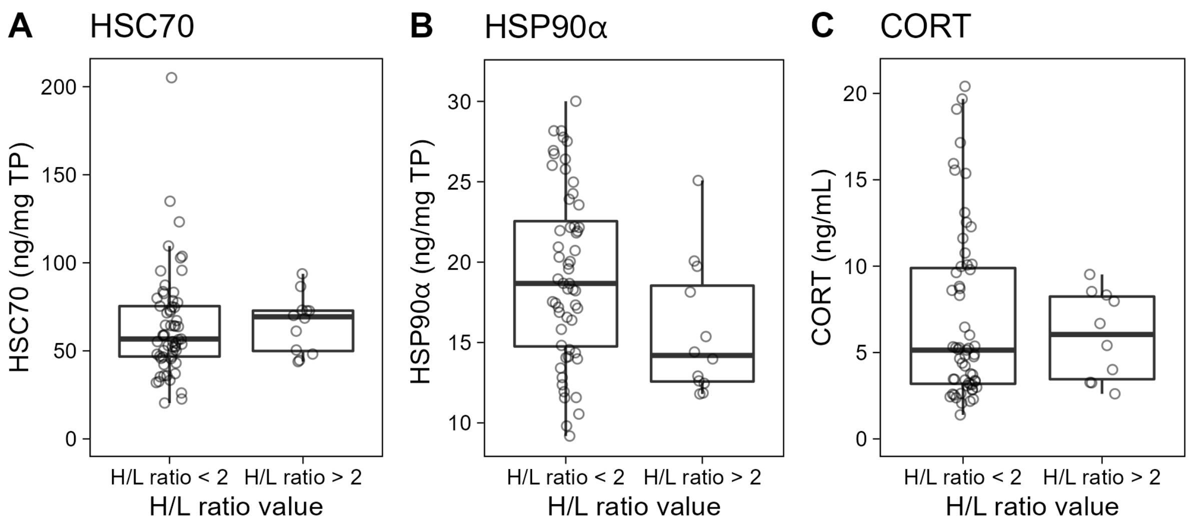

Figure S2. Boxplots of (A) HSC70, (B) HSP90α and (C) CORT levels in nestlings having an H/L ratio below or above 2 (considered abnormally elevated, not due to experimental conditions). Boxplots represents first and third quartiles, the horizontal line shows the median, and the whiskers extend to the 1.5*inter-quartile range. Open circles show individual data.
